# Assessment of cardiac remodeling in asymptomatic mitral regurgitation for surgery timing: a comparative study of echocardiography and magnetic resonance imaging

**DOI:** 10.1186/1476-7120-8-32

**Published:** 2010-08-13

**Authors:** Oner Ozdogan, Alper Yuksel, Cemil Gurgun, Meral Kayikcioglu, Oguz Yavuzgil, Cahide S Cinar

**Affiliations:** 1 Tepecik Training and Research Hospital, Cardiology Department, Izmir, Turkey; 2 Kent Hospital, Radiology Department, Izmir, Turkey; 3 Ege University, Cardiology Department, Izmir, Turkey

## Abstract

**Background:**

Early surgery is recommended for asymptomatic severe mitral regurgitation (MR), because of increased postoperative left ventricular (LV) dysfunction in patients with late surgery. On the other hand, recent reports emphasized a "watchful waiting" process for the determination of the proper time of mitral valve surgery. In our study, we compared magnetic resonance imaging (MRI) and transthoracic echocardiography to evaluate the LV and left atrial (LA) remodeling; for better definitions of patients that may benefit from early valve surgery.

**Methods:**

Twenty-one patients with moderate to severe asymptomatic MR were evaluated by echocardiography and MRI. LA and LV ejection fractions (EFs) were calculated by echocardiography and MRI. Pulmonary veins (PVs) were measured from vein orifices in diastole and systole from the tangential of an imaginary circle that completed LA wall. Right upper PV indices were calculated with the formula; (Right upper PV diastolic diameter- Right upper PV systolic diameter)/Right upper PV diastolic diameter.

**Results:**

In 9 patients there were mismatches between echocardiography and MRI measurements of LV EF. LV EFs were calculated ≥60% by echocardiography, meanwhile < 60% by MRI in these 9 patients. Severity of MR evaluated by effective regurgitant orifice area (EROA) didn't differ with preserved and depressed EFs by MRI (p > 0.05). However, both right upper PV indices (0.16 ± 0.06 vs. 0.24 ± 0.08, p: 0.024) and LA EFs (0.19 ± 0.09 vs. 0.33 ± 0.14, p: 0.025) were significantly decreased in patients with depressed EFs when compared to patients with normal EFs.

**Conclusions:**

MRI might be preferred when small changes in functional parameters like LV EF, LA EF, and PV index are of clinical importance to disease management like asymptomatic MR patients that we follow up for appropriate surgery timing.

## Introduction

In chronic mitral regurgitation (MR), symptoms do not develop until decompensation of the left ventricle (LV). Patients with MR, who are asymptomatic with normal ventricular performance, will need valve surgery at an annual rate of 10.3% [1] and early surgery is recommended for asymptomatic severe MR because of increased postoperative LV dysfunction [2] in patients with delayed surgery. However, recent reports have emphasized another process called "watchful waiting" [3] for relatively low-risk patients.

Mortality after delayed valve surgery is significantly increased with severe MR (Effective Regurgitant Orifice Area (EROA) ≥0.4 cm^2^) [4] and depressed LV ejection fraction (EF < 60%). Therefore, timing of the valve surgery for asymptomatic MR should be based on quantitative grading of the regurgitation severity and assessment of LV systolic dysfunction by transthoracic echocardiography [5].

Cardiac magnetic resonance imaging (MRI) is a superior method in evaluating EFs, LA volumes, and pulmonary veins (PVs) [6,7]. Enlarged LA volumes and PVs are important components of clinical deterioration in patients with MR; including atrial fibrillation and these could be subtle markers of early deterioration in asymptomatic patients with moderate to severe MR. In the present study, we compared cardiac MRI and transthoracic echocardiography to evaluate the concealed remodeling in LV, LA, and PV; for better definitions of patients that may benefit from early valve surgery.

## Methods

### Study population

Forty-six patients who had been under medical follow-up for moderate to severe MR in our outpatient clinic were evaluated by transthoracic echocardiography. Patients were excluded if they had MR due to ischemic heart disease and if they had an LV EF < 60% by echocardiography. Associated valve diseases such as mitral stenosis, aortic or tricuspid valve disease and arrhythmia (including sinus tachycardia) were also accepted as exclusion criteria. After these exclusion criteria, remaining 38 patients were assessed with respect to New York Heart Association (NYHA) functional class. Asymptomatic MR was defined as having functional capacity of class I or I-II according to patient's history (NYHA Class ≤I-II) and physical examination. Finally, 21 patients with asymptomatic MR were included to the study analysis. These 21 patients underwent transthoracic echocardiography and MRI for further cardiac evaluation. Before MRI was performed, all patients were reevaluated for their NYHA functional Class and physical examination findings like hypertension, tachycardia, crepitan ralles and edema; to minimize the hemodynamic dependent differences. Study protocol was approved by the local ethics committee and written informed consent was obtained from all participants.

### Echocardiographic Measurements

All patients were evaluated by Sonos 7500 ultrasound machine (Philips) equipped with 2.5 MHz transducer. Two-dimensional and Doppler flow parameters were measured according to American Society of Echocardiography recommendations [8]. LA volume was calculated using the area-length technique [9,10] from 4-chamber and 2-chamber views. LV diameters, volumes, and EFs were measured as recommended [11]. All images were analyzed on two occasions by two independent cardiologists; inter-observer correlation (*rho*) for maximum LVEF was 0.95 (p < 0.001) and for LA volume was 0.94 (p < 0.001).

The etiology of MR was determined according to subvalvular apparate and mitral valve morphology. The proximal isovelocity surface area was determined by measuring proximal-flow convergence [12]. Patient with an EROA >0.4 cm^2 ^was accepted as having severe MR, and >0.2 cm^2 ^as moderate MR [13]. Standard LV diastolic inflow was obtained in apical 4-chamber view by placing the sample volume at the valve tips level. Peak early inflow velocity (E) (m/s), peak atrial inflow velocity (A) (m/s), and their ratio were determined. Pulmonary vein (PV) flow velocities were obtained by positioning the sample volume into the right upper PV approximately 1 cm above its entry into the LA. Peak systolic (S) and peak diastolic (D) PV flow velocities were measured and S/D ratio was calculated. PV flow reversal duration and the velocity during atrial contraction were determined [14].

### Cardiac Magnetic Resonance Imaging

**Figure 1:**
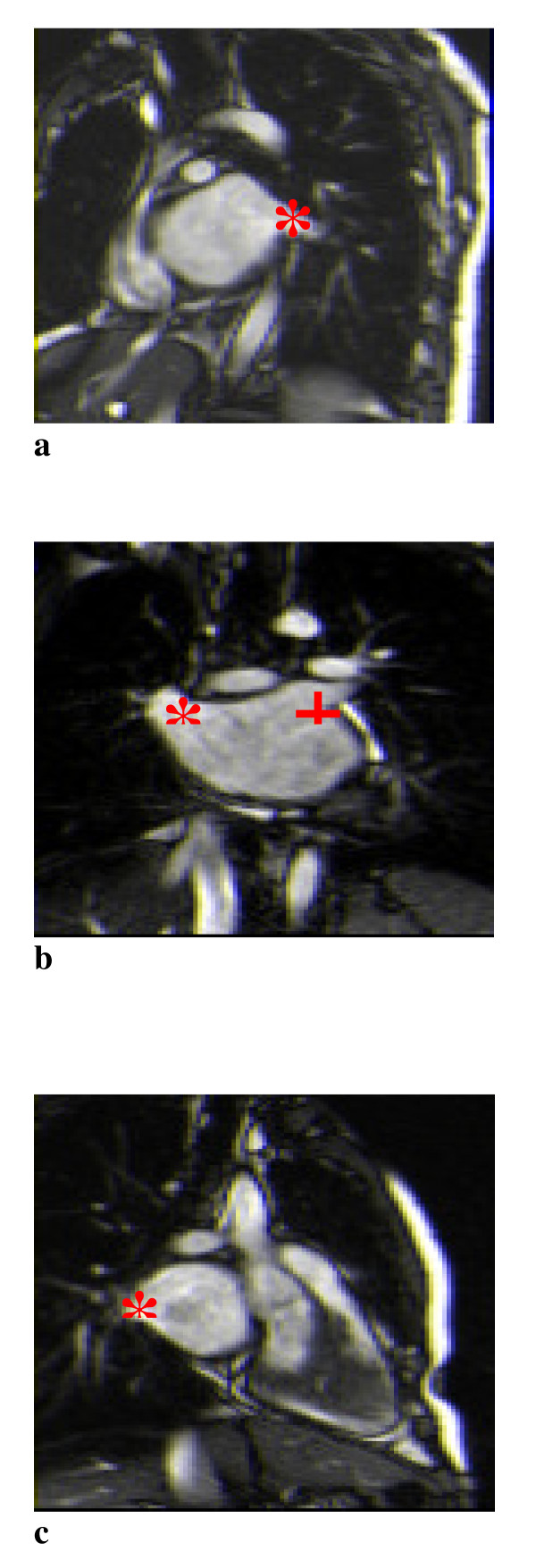
**Demonstration of four pulmonary veins**. 1a *Left lower pulmonary vein. 1b *Right upper pulmonary vein. +Left upper pulmonary vein. 1c *Right lower pulmonary vein.

**Figure 2:**
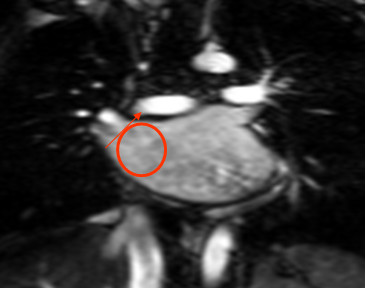
**Measurement of right upper pulmonary vein index**. Right upper pulmonary vein index: PV diastolic diameter-PV systolic diameter. PV diastolic diameter.

All MRI studies were acquired with a 1.5-T MRI scanner (Siemens Magnetom, Shymphony, Erlangen) and evaluated in ARGUS cardiac software (Siemens). For the evaluation of cardiac functions; images were acquired in standard planes, which were positioned either parallel to (horizontal and vertical long-axis planes) or perpendicular to the long axis of the heart (short-axis planes) [15]. We evaluated MR with standard cardiac planes obtained with ECG triggering cine gradient echo (GRE) sequences (FLASH, TrueFISP). Phase images were obtained by mitral FLASH technique. LV was scanned by consecutive sections at four chambers, long-axis, and short-axis by cine MRI. LA volumes, LA EFs, and LV EFs were measured with ARGUS software from the acquired images. LA EFs were calculated in 20 patients by MRI with the following formula: (LA end-diastolic volume-LA end-systolic volume)/LA end-diastolic volume. Axial and coronal planes were scanned at 6 mm sections by ECG-gated TrueFISP sequence to identify anatomic structure of PVs because of the connection and size variations. After determining PV connections at these images, all PVs [right upper PV, right lower PV, left upper PV, left lower PV] were displayed separately by breathold ECG-gated TrueFISP sequence with 5 mm section thickness (Figure 1). Because of breath disparities; when these sections did not pass through PVs properly, we used gap function (shifting the sections forwards and backwards only as thick as the interslice gap) to observe better images of PVs. When crosswise right upper-left lower PVs or left upper-right lower PVs were displayed at the same plane; if we considered the image was adequate, we didn't obtain additional images. To standardize these measurements, an imaginary circle that completed LA wall was drawn and right upper PVs were measured from the tangential of this circle (Figure 2). These measurements were repeated at the end of both systole and diastole. Right upper PV indices were calculated in 19 patients with the following formula: (Right upper PV diastolic diameter- Right upper PV systolic diameter)/Right upper PV diastolic diameter as a sign of atrial compliance. Right and LV volumes were assessed by ECG-triggered cine sequence and breathhold technique. The contours were drawn manually. The relationship between the severity of MR and PV index was assessed. LA EFs were also evaluated in patients with both depressed and normal LV EFs. Variability between the measurements of MRI was evaluated in 12 randomly selected subjects twice by the same observer and by two independent observers for interobserver and intraobserver variabilities. The measurements by two independent observers and by the same observer at different times did not differ in statistically significant terms (p > 0.05).

### Statistical Analysis

**Figure 3:**
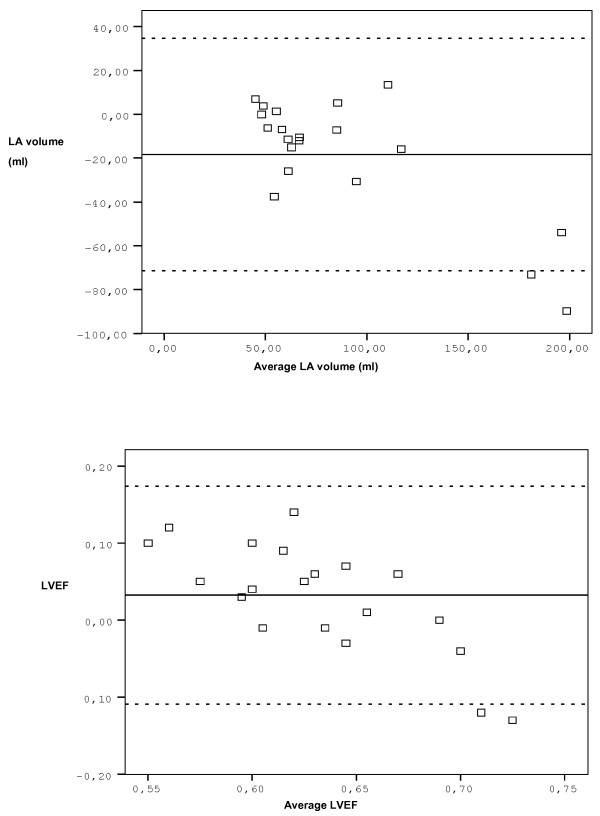
**Bland Altman analysis of LA and LVEF measured by echocardiography compared to MRI is shown in (a) and (b) respectively**. Average difference (solid) is shown along with 95% limits of agreement (dashed).

Statistical analysis was performed with SPSS 13.0 software. All values were expressed as mean ± standard deviation. Association between PV indices and echocardiographic parameters were evaluated with Pearson and Spearman correlation analysis. P value less than 0.05 was considered as statistically significant. Independent sample t test was performed to compare means of variables of groups to determine the statistical significance of difference. The relationship between echocardiograhic measurements and MRI measurements was analyzed by linear regression and Bland Altman analysis (Figure 3).

## Results

Baseline characteristics of the patients are shown in Table 1. The etiology of MR was rheumatic valve disease in 13 patients and mitral valve prolapse in 6 patients, whereas both etiologies were observed only in 2 patients. None of the patients had accompanying valve disease except for mild tricuspid valve regurgitation in 10 patients. Qualitatively assessed MR severity was third grade in twelve patients. All of the patients were in regular sinus rhythm with heart rate of 60-90/min. The mean systolic and diastolic blood pressure values measured before echocardiography and MRI were not statistically different (122.3 ± 8.9, 77.9 ± 5.1 and 127.7 ± 8.8, 78.0 ± 5.0, respectively, p > 0.05).

**Table 1:** Characteristics of Patients with Asymptomatic Mitral Regurgitation (n:21)*

Characteristics	
Age (y)	44 ± 16*
Female gender n (%)	15 (71)
Functional capacity, n (%)	
*NYHA Functional Class I*	13 (61)
*NYHA Functional Class I-II*	8 (38)
Drugs, n (%)	
*ACE inh.*	3 (14)
*Beta-Blocking agent*	3 (14)
Mitral regurgitation follow-up n (%)	
*0-6 month*	3 (14)
*6 months-1 year*	3 (14)
*1 year< x < 3 years*	7 (33)
*3 year< x < 6 years*	2 (10)
*>6 years*	6 (29)

* Plus-minus values are means ± SD

† NYHA: New York Heart Association, ACE inh.: Angiotensin Converting Enzyme Inhibitors

**Figure 4:**
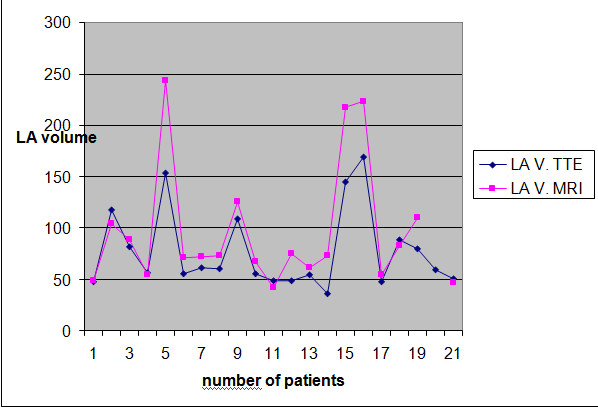
**Comparison of left atrial volumes calculated by echocardiography and cardiac magnetic resonance imaging**. LA V: Left atrial volume. TTE: Transthoracic echocardiography. MRI: Magnetic resonance imaging.

Table 2 demonstrates the echocardiographic findings of the study population. None of the patients had systolic pulmonary artery pressure over 40 mmHg. Mean LA EF calculated by MRI was 0.27 ± 0.13. Figure 3 shows the agreement between echocardiography and MRI. The differences between the two methods were not statistically significant for LA volume or LV EF. LA volumes calculated by cardiac MRI were larger than LA volumes calculated by echocardiography; however the differences were not statistically significant (p > 0.05) (Figure 4). There were positive correlations between LA volumes calculated by echocardiography and cardiac MRI (r: 0.940, p < 0.0001). There was a negative relationship between EROA and LA EF, but did not reach statistically significant level (r:-0.412, p: 0.071).

**Table 2:** Echocardiographic data of patients with mitral regurgitation (n:21)

Echocardiographic data	Mean	(min-max)
Left atrium (cm)	4.2 ± 0.7	(3.0-6.0)
Right ventricle (cm)	2.3 ± 0.3	(1.7-2.8)
Left ventricle end-diastole (cm)	4.9 ± 0.4	(4.4-5.9)
Left ventricle end-systole (cm)	3.0 ± 0.4	(2.4-3.8)
Interventricular septum (cm)	1.0 ± 0.1	(0.8-1.3)
Posterior wall (cm)	1.0 ± 0.1	(0.9-1.3)
Mitral E wave velocity (cm/s)	108 ± 28	(67.3-190.0)
Mitral A wave velocity (cm/s)	91.2 ± 30.2	(48.0-156.0)
E/A ratio	1.2 ± 0.3	(0.7-1.7)
Deceleration time (ms)	212 ± 51	(125-300)
Isovolumic relaxation time (ms)	87 ± 32	(55-150)
Left atrial area 1 (cm^2^)	21.6 ± 6.3	(13-33)
Left atrial area 2 (cm^2^)	20.7 ± 6.8	(12-36)
Left atrial volume (cm^3^)	77.3 ± 38.7	(36-169)
Pulmonary vein S wave velocity (cm/s)	58.5 ± 11.0	(45-84)
Pulmonary vein D wave velocity (cm/s)	52.8 ± 13.0	(27 -82)
Pulmonary vein A wave velocity (cm/s)	37.2 ± 9.5	(26.4-64.7)
Pulmonary vein A wave duration (ms)	107.5 ± 18.4	(85-150)
Mitral regurgitation velocity (cm/s)	524.1 ± 98.9	(266-662)
Left ventricle ejection fraction (%)	60 ± 3	(60-70)
SPAP (mmHg)	24.8 ± 5.5	(18-33)

A: Atrial, D: Diastolic, S: Systolic, Left atrial area 1: From four chamber view, Left atrial area 2: From two chamber view, SPAP: Systolic pulmonary artery pressure

The mean LV EF values measured by echocardiography and MRI were not statistically different (0.64 ± 0.03 and 0.61 ± 0.08, respectively, p > 0.05). However, in 9 patients there were mismatches between LV EFs calculated by these two techniques. In these 9 patients, LV EFs were determined moderately decreased (<60%) by cardiac MRI. Besides, there were no significant differences in EROAs between patients with normal and depressed EFs determined by MRI (0.27 ± 0.13 cm^2 ^vs. 0.28 ± 0.18 cm^2^, p > 0.05). LA EFs were also significantly reduced in patients with depressed LV EFs by MRI (0.19 ± 0.09 vs. 0.33 ± 0.14, p: 0.025) (Table 3).

**Table 3:** Comparison of echocardiography and magnetic resonance imaging (MRI) data according to left ventricle ejection fractions (LV EFs)

	Patients with preserved LV EF (≥60%) in TTE and depressed EF(<60%) in MRI	Patients with preserved LV EF (≥60%) both in TTE and MRI	p
n	9	12	
Age (yrs)	50 ± 15	40 ± 16	0.17
LA volume (cm^3^)	86.1 ± 47.36	70.36 ± 31.42	0.67
EROA (cm^2^)	0.27 ± 0.13	0.28 ± 0.18	0.8
CFA (cm^2^)	7.38 ± 2.62	7.20 ± 4.17	0.65
LA EF	0.19 ± 0.09	0.33 ± 0.14	0.025
RUPV index	0.16 ± 0.06	0.24 ± 0.08	0.024

CFA: color flow area, EF: ejection fraction, EROA: effective regurgitant orifice area, LA: left atrium, LV: left ventricle, MRI: magnetic resonance imaging, RUPV: right upper pulmonary vein, TTE: transthoracic echocardiography.

There was no correlation between right upper PV indices and the severity of MR. According to LV EF, right upper PV indices were found lower in nine patients who were supposed to be late for mitral valve surgery (EF < 60%), in comparison with patients with preserved EFs (0.16 ± 0.06 vs. 0.24 ± 0.08, p: 0.024). There was no significant correlation between the velocities measured with pulse wave Doppler from the right upper PV (including systolic, diastolic velocities and the proportion of systolic-diastolic flow velocities: S/D) and right upper PV index and the other PV indices (p > 0.05).

## Discussion

Despite the increasing tendency for early surgery in the management of asymptomatic severe MR, watchful waiting is a notable option for some selected patients [3]. Therefore, it is important to differentiate the subgroups of asymptomatic patients with MR that early surgery should be considered. This study was designed to find out if cine MRI could give us more information to evaluate this subgroup, i.e. to define patients in this group who might have irreversible changes in LV and LA that couldn't be detected by echocardiography at the time of evaluation for surgery decision.

According to the published guidelines; moderately reduced LV EF (<60%) is an indicator of mitral valve surgery decision in MR [5]. Echocardiography could sometimes fail in calculating the LV EFs, because of the 3-dimentional enlargement of the ventricle. Similarly; in 9 patients (42%); LV EFs were moderately depressed (<60%) by MRI, while all LV EFs were preserved (≥60%) by echocardiographic evaluation. The values of LV EFs (<60%) published on guidelines [5] are based on echocardiographic results and not on MRI results and for that reason maybe specific LV EF threshold values by MRI should be determined as indicators for mitral valve surgery timing. Lower LA EFs in these 9 patients could also indicate the ongoing remodeling including both LV and LA, despite watchful waiting. Additionally, reduced right upper PV indices might be the indicators of clinical deterioration including atrial fibrillation. Therefore, MRI should be preferred when small changes in functional parameters are of clinical importance to disease management like asymptomatic MR that we follow up for appropriate surgery timing [7,16].

The advantages of MRI over echocardiography are emphasized by Bellenger at al. as better image quality and quantification possibilities with high reproducibility that requires smaller sample sizes to prove statistical significance [17]. MRI was suggested to be an accurate and reproducible method in patients with heart failure that yields serial assessment of LV remodeling [18]. Westenberg et al. searched restrictive annuloplasty results by MRI. They demonstrated significant LA and LV reverse remodeling over time using MRI [19]. Since follow-up studies performed by echocardiography are not optimal for precise assessment of LA and LV volumes, MRI was considered to be the gold standard method for assessment of LV function and volumes [20]. On the other hand, LA volume measurements in MR images were also shown to be reproducible in clinical practice according to previous studies [21] and MRI has become the gold standard for LA volume determination [22-25].

History of mitral valve disease duration sometimes does not reflect the exact period, because of the long asymptomatic clinical course of chronic MR. Hence we defined a new index; PV index that could help to define both mitral valve disease duration and the severity. All four PVs could be displayed by MRI and their indices could be calculated as the components of LA remodeling. In our study, right upper PV diameters were measured both in diastole and systole by MRI and right upper PV indices for each patient were calculated. In nine patients who were supposed to be late for mitral valve surgery according to LV EFs (LV EF < 60%), right upper PV indices were found lower by MRI. Although PV index does not give information about MR severity; it might reflect the remodeling of LA. LA remodeling is a consequence of the duration of MR and its severity, therefore it is an important determinant of hemodynamic and clinical picture in patients with MR. In a published study, the comparison of 3 D magnetic resonance angiography with 2 D cine MRI for characterizing anatomy and size of PV revealed that these two methods were similar with regard to the evaluation of PVs. In that study, reduced difference between the PV diameter at systole and diastole was observed in patients with atrial fibrillation [26]. Accordingly, reduced right upper PV index may alert us for a new atrial fibrillation that originates from PVs and points out a relatively high-risk subgroup of asymptomatic MR. It is important for watchful waiting process with MR. After atrial fibrillation, patients become symptomatic and a new stage begins with a rapid deterioration of the disease. As this index is defined for the first time, its thresholds are not clear yet, but we believe that, randomized clinical trials with large study populations can determine the thresholds for PV index.

## Limitations

The most important limitation of our study is the small sample size because of using too many exclusion criteria that could affect our study results. Age was not considered as eligibility criteria; however age could be an important factor for PV indices and LA remodeling. PV index is a novel parameter and a preliminary validation study could be helpful for this index. Our study could be improved by following these patients for long-term mortality and morbidity, but it was not ethical to observe patients with severe MR and reduced EF determined by MRI. Therefore, mitral valve surgery was recommended for these 9 patients with depressed ejection fractions by MRI.

## Conclusion

In asymptomatic patients with MR, the most important issue is the decision of valve surgery. Despite the new approaches like watchful waiting process, early surgery is recommended when valve repair is easy and feasible. Evaluating asymptomatic MR by MRI is important for more favorable calculation of LV EF. Reduced LA EFs and right upper PV indices by MRI, as signs of LA remodeling might be the predictors of worsening clinical outcome of asymptomatic MR; including atrial fibrillation. We believe that, MRI should be preferred when small changes in functional parameters like LV EF, LA EF, and PV index are of clinical importance to disease management like asymptomatic MR that we follow up for appropriate surgery timing [7,16].

## Consent

All patients provided written informed consent to participate in the research.

## Competing interests

The authors declare that they have no competing interests.

## Authors' contributions

OO carried out the study, made echocardiographic examinations and drafted the manuscript. AY performed magnetic resonance imaging studies. First two authors were equally contributed to the study. MK performed the statistical analysis. CG and OY participated in the design of the study. CSC participated in study design and coordination. All the authors read and approved the manuscript. None of the authors have conflict of interest in any of the material mentioned in the manuscript.
